# A novel DNA-binding feature of MeCP2 contributes to Rett syndrome

**DOI:** 10.3389/fncel.2013.00064

**Published:** 2013-05-09

**Authors:** Xin Xu, Lucas Pozzo-Miller

**Affiliations:** Department of Neurobiology, The University of Alabama at BirminghamBirmingham, AL, USA

Rett syndrome (RTT) is an X-linked neurodevelopmental disorder associated with intellectual disabilities, which almost exclusively affects females during early childhood with an incidence of 1:10,000–15,000 worldwide (Neul and Zoghbi, 2004). RTT is primarily caused by loss-of-function mutations in methyl-CpG-binding protein 2 (*MECP2*) (Amir et al., 1999), the gene encoding MeCP2, a transcriptional repressor that binds to methylated CpG sites in promoter regions of DNA (Lewis et al., 1992; Nan et al., 1997). But to date, effective treatments for RTT remain lacking, which makes the identification of critical MeCP2 function of great importance to decipher the molecular mechanisms of RTT pathogenesis. Recently, Baker et al. (2013) identified a highly conserved AT-hook domain important for MeCP2 function and closely related to clinical progressions observed in RTT.

**Figure 1:**
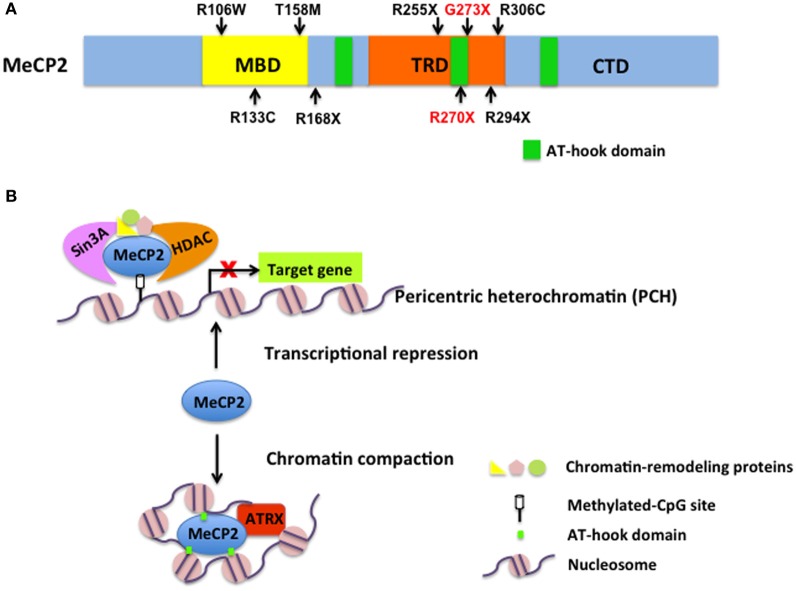
**MeCP2 structure and function in gene regulation and chromatin remodeling. (A)** A schematic diagram of MeCP2 structure with functional domains and three AT-hook domains. Eight common MeCP2 mutations causing RTT and the two mutants discussed by Baker et al. (2013) are indicated in black and red, respectively. Note that R270X is also one of the most common mutations found in RTT individuals. **(B)** The function of MeCP2 in transcription repression and chromatin compaction. As a transcriptional repressor, MeCP2 recruits Sin3A, histone deacetylases (HDACs), and some chromatin-remodeling proteins to silence target gene transcription. Also, Baker et al. (2013) discovered that AT-hook clusters facilitate MeCP2 to alter chromatin conformation by recruitment of ATRX to PCH to maintain chromatin homeostasis.

MeCP2 is an abundant chromatin-associated nuclear protein with high affinity binding to DNA containing methyl-CpG throughout mammalian genomes (Lewis et al., 1992; Nan et al., 1997). MeCP2 contains two well-defined functional domains: an N-terminal methyl-CpG binding domain (MBD) (Figure 1A), essential for its selective binding to 5-methylcytosine; and a central transcriptional repression domain (TRD) (Figure 1A) that recruits the transcriptional co-repressor Sin3A, histone deacetylases (HDACs) (Jones et al., 1998; Nan et al., 1998) and other related chromatin-remodeling proteins (Chahrour and Zoghbi, 2007) (Figure 1B). In addition, a C-terminal domain (CTD) (Figure 1A) facilitates DNA binding and protein–protein interactions (Chandler et al., 1999; Buschdorf and Stratling, 2004). Sporadic mutations in *MECP2* cause 95% cases of RTT, among which eight specific ones are the most common and account for >65% of all individuals with RTT (R106W, R133C, T158M, R168X, R255X, R270X, R294X, R306C) (Figure 1A) (Calfa et al., 2011). Several phenotype–genotype correlation studies have contributed to gaining insight into the role of MeCP2 in brain development. In the February 28, 2013 issue of *Cell*, Baker and colleagues describe the generation of two new mouse lines bearing either a R270X or G273X truncation to mimic male RTT patients with R270fs and G273fs mutations, respectively. Characterizing these mice, they discovered that G273X mice exhibit a significantly delayed disease progression with a longer lifespan compared to R270X mice, which led to the hypothesis that the region between R270 and G273 is important for MeCP2 function.

How is that just 3 amino acids, have such significant consequences for the progression and severity of the mouse RTT-like phenotype? Using chromatin immunoprecipitation followed by sequencing (ChIP-seq), the authors found that both MeCP2-R270X and MeCP2-G273X bind to DNA globally, very much the same as wildtype MeCP2 does. Then, they focused on the TRD (amino acids 207–310) where, these two mutations locate, and found that both mutations disrupt normal repressor activity of MeCP2. By looking more closely at gene expression over the course of disease, the authors discovered a delay in misregulation of a small subset of genes in G273X mice, although most of those genes eventually become misregulated as they do in R270X mice. Despite that MeCP2's repressor activity is disrupted in G273X mice, these mice lived longer and healthier than *Mecp2* KO mice, which suggests additional functions.

A highly conserved AT-hook domain in MeCP2 that terminates at G273 (Figure 1A) answers the question. Adrian Bird's group had already described an AT-hook domain in MeCP2, but its function was unclear (Klose et al., 2005) (Figure 1A). AT-hooks are regions of a protein that bind to AT-rich DNA. MeCP2 requires an A/T-rich motif adjacent to methylated CpG dinucleotides for efficient DNA binding (Klose et al., 2005). Using an electrophoretic mobility shift assay (EMSA), the authors discovered a key difference between G273X and R270X mutants: loss of function of the second AT-hook domain impairs the DNA-binding ability of the R270X mutant, indicating that amino acids 270–272 are essential for the DNA-binding feature of MeCP2. However, it is unclear if the probe used in the EMSA contained A/T-rich motives, which raises caution when considering this new MeCP2 feature as its most critical in transcriptional regulation. Prior work had shown the TRD-CTD domain of MeCP2 is largely responsible for facilitating oligomerization of nucleosomal arrays, as well as chromatin compaction (Ghosh et al., 2010). Using an *in vitro* assay of chromatin compaction, Baker et al. (2013) show that MeCP2-R270X does not compact chromatin as efficiently as MeCP2-G273X does. These results not only provide clear evidence that this second AT-hook domain plays an important role in manipulating chromatin structure, but also explain the phenotypic differences observed in G273X and R270X mice. The authors propose that, after the MBD of MeCP2 binds to methylated DNA regions, the AT-hook clusters participate in altering DNA conformation to aid in chromatin packing (Figure 1B).

Furthermore, Baker and colleagues showed that the localization of α-thalassemia/mental retardation syndrome X-linked protein (ATRX) to pericentric heterochromatin (PCH) is a distinguishing feature between G273X and R270X. This is an intriguing finding not only because ATRX syndrome shows overlap with RTT, but also because ATRX participates in chromatin remodeling, associates with MeCP2, and disruption of that interaction is thought to contribute to intellectual disability (Nan et al., 2007). The new findings by Baker et al. (2013) strongly suggest that ATRX contributes to the function of MeCP2 on chromatin modification (Figure 1B). It would be interesting to further explore the role of ATRX in MeCP2 dysfunction not only in RTT individuals, but also in other intellectual disabilities associated with *MECP2* mutations.

In summary, Baker et al. (2013) identified a novel AT-hook domain of MeCP2 that plays an important role in chromatin organization, providing a new model involving an additional protein partner, which, incidentally, is also implicated in a neurodevelopmental disorder associated with intellectual disability. They took advantage of the correlation between mouse models and human individuals, identified the pathogenesis of various RTT-like phenotypes in novel mouse lines, which increased our understanding of how different MeCP2 functions are affected by various disease-causing mutations. In this regard, we very much share the hopes of the authors that their discovery of a new fundamental feature of MeCP2 can further help in developing novel therapeutic approaches for RTT and other intellectual disabilities associated with *MECP2* mutations.
